# The tortuous diagnosis of one case of neonatal hyperthyroidism

**DOI:** 10.1186/s12887-024-04531-6

**Published:** 2024-01-13

**Authors:** Lin Zhu, Jing Wang, Wei Liu

**Affiliations:** Department of Pediatrics, Tongji Hospital, Tongji Medical College, Huazhong University of Science and Technology, No. 1098 Jiefang Avenue, Wuhan, Hubei 430077 P.R. China

**Keywords:** Hyperthyridism, Newborn, Hepatic lesion, Misdiagnose, Prognosis

## Abstract

**Objective:**

To outline the clinical signs, diagnosis, and course of care for a single case of neonatal hyperthyroidism while also summarizing common diagnostic errors related to this condition.

**Methods:**

Medical records of the neonate of hyperthyroidism were collected and analyzed in combination with literature.

**Results:**

The neonate’s mother had thyroid disease, but her thyrotropin receptor antibody (TRAb) levels were not monitored during pregnancy. The neonate exhibited typical symptoms of hyperthyroidism on the day of birth but was not diagnosed until 15 days later. Impaired liver (cholestasis, elevated liver enzymes) and cardiac function (pulmonary hypertension, right heart enlargement) are the main manifestations. Treatment with methimazole (1.0 mg /kg·d) and propranolol (2.0 mg /kg·d) led to recovery, and the neonate stayed in the hospital for 27 days before being discharged with medication. The diagnosis was temporary hyperthyroidism, and the medication was discontinued at 72 days of age.

**Conclusion:**

It is important to strengthen the management of high-risk pregnant women with thyroid disease. Monitoring TRAb levels in both mothers and neonates should be done dynamically to enable early prediction and diagnosis of neonatal hyperthyroidism. Most neonates with hyperthyroidism have a good prognosis when timely and appropriate medical treatment is provided.

## Impact

The tortuous diagnosis processes of one case of NH.

In the absence of specific clinical manifestations of NH, how to quickly diagnose it.

Reminding clinicians of the importance of early identification of NH, as well as the management of pregnant women with abnormal thyroid function.

## Introduction

Neonatal hyperthyroidism (NH) is an uncommon condition that affects 1–5% of newborns born to mothers who have active or past Graves’ disease (GD) [1], and it affects 22% of pregnant women who need to take long-term antithyroid drugs (ATDs) treatment. Autoimmune NH is related to the transplacental passage of maternal anti-thyrotropin receptor antibodies (TRAbs) [2]. The fetal thyroid gland becomes responsive to thyroid-stimulating hormone (TSH) and TRAbs at around 20 weeks of gestation [3], those antibodies stimulating the fetal thyroid, cause in-utero and/or postnatal hyperthyroidism. Fetal hyperthyroidism can cause goiter, heart failure with nonimmune hydrops, advanced bone maturation, intrauterine growth retardation, preterm birth, and even fetal death [4]. When fetal hyperthyroidism is present, there is a high probability of neonatal thyrotoxicosis, which is usually temporary and goes away in 4 to 6 months after birth following clearance of maternal TRAbs. Signs and symptoms of neonatal hyperthyroidism include goiter, tachycardia, poor feeding, irritability, tremors, sweating, and difficulty sleeping [5]. There are occasional cases of proptosis, craniosynostosis, and microcephaly. Without prompt treatment with antithyroid drugs, cardiac failure and death may occur [6].

At present, there are only more than 50 cases of NH reported in China. Due to the diversity and lack of specificity of the clinical manifestations of the disease, clinicians lack understanding of the disease, which is easy to be overlooked or misdiagnosed [7]. To improve knowledge of early identification and treatment of NH, our study presented one case of NH with prominent clinical signs of liver and cardiac dysfunction, whose mother had received GD therapy with radioactive iodine four years prior.

## Materials and methods

### Patient and clinical data

This study analyzed one newborn with confirmed hyperthyroidism who was hospitalized in Tongji Hospital, Tongji Medical College, Huazhong University of Science and Technology and has been discharged. The study was appproved by the Tongji Hospital ethics committee. Informed consent was obtained from the patient’s parents.

### Data collection

Clinical, and laboratory data, as well as therapeutic medication and clinical outcomes, were obtained from the electronic medical records.

### Definitions

The enrolled newborn in this study was diagnosed with hyperthyroidism, according to the “Practice of Neonatology, 5th Ed”.

The diagnostic criteria of NH were as follows: (1) The mother had a history of autoimmune thyroid disease, especially hyperthyroidism. (2) The newborn had typical symptoms and signs [8]: excitement, irritability, tremors; skin flushing, sweating; increased appetite, accompanied by vomiting and diarrhea, and unsatisfactory weight gain; he or she likes opening eyes, periorbital edema, eyelid contracture, and exophthalmos; goiter may be present; increased heart rate and respiration, hypertension, enlargement of liver and spleen, etc. Severe cases may have a fever, arrhythmia, heart failure and jaundice, liver failure, and coagulation disorders. (3) With laboratory examination: serum T3, and T4 increased, and TSH decreased. This neonate met the diagnostic criteria of NH.

## Results

### Patient history and clinical features

The neonate was delivered prematurely at 32^+ 6^ weeks of gestation as the first offspring of unrelated parents. Her weight measured 2220 g (80%), length measured 47.5 cm (97%) and head circumference measured 28.5 cm (20%). Due to exhibiting symptoms of “moaning and foaming” immediately after birth, she was admitted to the Neonatal Intensive Care Unit (NICU). After admission, the neonate’s blood oxygen saturation was 65% without oxygen inhalation, and her shin wascyanotic, so CPAP (Continuous Positive Airway Pressure) assisted ventilation was given, but her dyspnea persisted after 4 h, and she developed “convulsion”. Consequently, invasive ventilator-assisted ventilation was initiated. The examination findings after admission are presented in Table 1, encompassing thrombocytopenia; elevated transaminase and total bilirubin, particularly direct bilirubin; hepatosplenomegaly, bile sludge formation; right heart enlargement, and the presence of pulmonary hypertension. The primary diagnoses are neonatal respiratory distress syndrome (NRDS) and intrauterine infection(?). In addition to respiratory support, administration of blood products, anti-infection agents, cardiac and hepatic protection, cholang-removing blood stasis, reduction of pulmonary artery pressure, and enhancement of circulation were implemented.

**Table 1:** The demographic, clinical and biochemical characteristics of the patient

Age (Days)	0	1	4	6	8	15	23	40	57	72	93	108
**Corrected gestational age (weeks)**	32^+ 6^	33	33^+ 3^	33^+ 5^	34	35	35^+ 6^	38^+ 2^	40^+ 5^	42^+ 6^	45^+ 6^	48
Weight (g)/%*	2220(80)	2200(77.8)	2020(69.5)	2090(46.1)	2120(46.5)	2060(23.3)	2100(13.1%)	2700(18.5)	NA	3600(24.9)	NA	5300(67.6)
Height (cm)/%*	47.5(97)	NA	NA	48(95.1)	NA	48(85.2)	49.6(90%)	50.1(69.7)	NA	55.5(92.2)	NA	NA
Head circumference (cm)/%*	28.5(20)	NA	NA	NA	NA	NA	NA	31.6(6.5)	NA	34.2(0.0)	NA	NA
**NBNA**	NA	NA	NA	NA	NA	NA	NA	36	NA	38	NA	NA
**Blood biochemical parameters**												
Leukocytes (3.5–9.5) 10^9^/ L	13.5	10.29	7.5	7.47	9	18.45	9.25	NA	NA	NA	NA	NA
Neutrophils (1.8–6.3) 10^9^/ L	10	6.29	3.94	3.2	3.89	7.97	3.37	NA	NA	NA	NA	NA
Hemoglobin (130–175 g/ L	187	177	190	173	170	167	142	NA	NA	NA	NA	NA
Platelets (125–350) 10^9^/ L	53	63	92	144	195	251	164	NA	NA	NA	NA	NA
Alanine aminotransferase (9–50) U/ L	86	95	80	94	137	212	175	105	77	93	21	NA
Aspartate aminotransferase (15–40) U/ L	366	298	273	334	331	355	232	109	63	71	46	NA
Total bilirubin (2-20.4) µmol/ L	149.3	177.1	279.1	326.4	315.6	258.5	173	108.7	42.3	22.2	6	NA
Direct bilirubin (2-20.4) µmol/ L	121.7	128	222.9	247.9	252.1	215.6	146.6	93.4	35.6	15.1	5.2	NA
C-reactive protein (0-0.5) mg/ dL	1.4	1.5	1.48	1.9	2.15	<0.5	<0.5					
TSH (0.27–4.2) uIU/mL						< 0.005	< 0.005	< 0.005	0.261	1.19	0.845	2.18
FT3 (3.1–6.8) pmol/L						6.2	5.92	2.68	3.07	3.26	4	3.92
FT4 (12–22) pmol/L						34.9	30.1	9.54	8.7	9.34	9.56	10
**Echocardiography**												
Transverse diameter of the right atrium (mm)	NA	16	15	NA	17	16	16	12	NA	NA	NA	NA
Transverse diameter of the right ventricle (mm) NA		16	16	NA	17	16	15	12	NA	NA	NA	NA
Tricuspid regurgitation	NA	severe	severe	NA	severe	severe	severe	moderate	NA	NA	NA	NA
Pulmonary artery pressure (mmHg)	NA	64	64	NA	64	60	44	37	NA	NA	NA	NA
Patent ductus arteriosus (mm)	NA	2.5	1.7	NA	1.7	1.4	1.6	NA	NA	NA	NA	NA
Patent foramen ovale (mm)	NA	2.3	2	NA	2	2	2	3	NA	NA	NA	
Ejection fraction (%)	NA	70	70	NA	64	67	67	70	NA	NA	NA	
**Abdominal ultrasonography**												
Distance between the right inferior border of the liver and the subcostal margin of the right midclavicular line (cm)	NA	4.7	4.8	NA	4.9	3.8	4.2	3.8	NA	NA	NA	
Spleen pachydiameter (cm)	NA	1.5	1.6	NA	1.5	1.3	1.2	1.7	NA	NA	NA	
Biliary sludge	NA	Yes	Yes	NA	Yes	Yes	Yes	No	NA	NA	NA	
Gallstone	NA	No	No	NA	No	No	Yes	Yes	NA	NA	NA	
**Treatment**												
Methimazole						→	→	→				
Propranolol						→	→	→				

*Percentile for children of the same gender and gestational age; NBNA: neonatal behavioral neurological assessment; NA: Not available.

### Diagnosis

After 2 weeks, the patient no longer requires oxygen assistance. However, there was no notable enhancement in liver and cardiac functionality. Additionally, the patient tended to open her eyes, an increased appetite but inadequate weight gain, and a rapid heart rate. Following consultation and pertinent examinations, we ruled out infection based on normal levels of inflammatory markers, negative results from blood culture, blood NGS-DNA, and a comprehensive panel of viral and other related pathogenic tests. Furthermore, inherited metabolic diseases were excluded based on normal levels of blood ammonia, lactic acid, pyruvate, blood amino acids, and urine organic acids. Lastly, rheumatic immune diseases were ruled out based on a negative complete set of rheumatism and the absence of any history of rheumatic immune system diseases in the patient’s family. We suspected that she had a genetic mutation-caused syndrome, so we advised the family to undergo comprehensive genetic testing. On the 15th day of her hospitalization, we conducted a routine screening for thyroid function and discovered that her TSH levels were below the normal range (TSH < 0.05uIU/mL), while FT4 levels were significantly elevated (34.9ng/dL) (Table 1). Additionally, her thyroglobulin levels were above 500 ng/ml (reference range: 3.5–77 ng/ml), thyroglobulin antibody levels were 15.6 IU/ml (reference range: <115 IU/ml), thyroid peroxidase antibody levels were 43 IU/ml (reference range: <34 IU/ml), and Thyrotropin receptor antibody levels were 29.7 IU per liter (reference value, < 1.75IU per liter). A thyroid ultrasound showed an increase in thyroid volume and abundant blood flow, leading to a diagnosis of NH.

A thorough examination of the neonate’s mother’s medical history was conducted: her thyroid function (11/26, 2019): TSH 0.01 uIU/mL, T3 14.63 pg/mL, T4 3.56 ng/dL, TPOAb 44 U/mL, TGAb < 15 U/mL. After Iodine 131 treatment, she has been treated with euthyrox orally until now, which further supported the neonate’s diagnosis of hyperthyroidism. Methimazole (0.5 mg/kg/d, Bid) and propranolol (1.8 mg/kg/d, Tid) were promptly added to the treatment regimen, and the neonate’s thyroid function was periodically reassessed. After taking methimazole and propranolol orally for 1 week, the levels of FT3 and FT4 decreased (Table 1). Additionally, the patient experienced a decrease in heart rate and an improvement in liver function (as indicated by decreased ALT, AST, and direct bilirubin levels), as well as a significant decrease in pulmonary artery pressure. The patient was discharged with medicine after 27 days of hospitalization.

### Follow-up examination

**Fig. 1:**
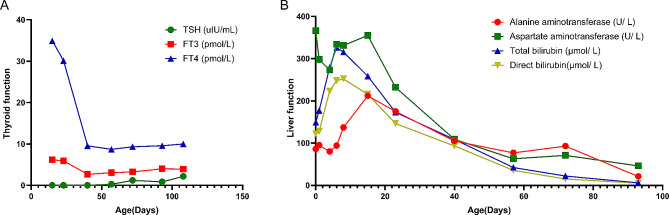
The thyroid function (**A**) and liver function (**B**) of the neonate were followed up with age

After 25 days of medication, the levels of FT3 and FT4 (except TSH) returned to the normal range (Table 1; Fig. 1A). Methimazole and propranolol were discontinued after 29 days and 36 days of oral medication, respectively. The patient’s thyroid function completely returned to normal 13 days after withdrawal of methimazole, subsequent intermittent reexaminations confirmed the maintenance of normal thyroid function.

With the improvement of thyroid function, catch-up growth of body weight and length was achieved. On day 72 after birth (postmenstrual age 42^+ 6^ weeks), the neonates’ weight and length were at the 24.9 percentile and 92.9 percentile for their sex and gestational age, respectively. The neonatal behavioral neurological assessment (NBNA) score was 38. Notably, the patient’s cardiac function, liver function (Fig. 1B), and hepatosplenomegaly showed significant improvement. During the whole treatment period of hyperthyroidism, no adverse reactions such as granulocytopenia or liver function damage were observed.

## Discussion

In this study, we report a typical case of NH with critical condition and impaired liver and cardiac function as the main clinical manifestations. The patient’s mother had been receiving oral euthyrox treatment since undergoing Iodine 131 radiotherapy for GD four years ago. However, TRAbs levels were not monitored during pregnancy. NH was diagnosed on the 15th day after birth. During the period, a multi-disciplinary consultation was conducted to screen for infectious diseases, inherited metabolic diseases, rheumatic immune diseases, and, other conditions. The diagnosis process was complex, providing valuable experience for future NH diagnoses.

The prevalence of hyperthyroidism in pregnancy ranges from 0.7 to 2.8% worldwide [9, 10], with GD as the most common etiology [11]. During pregnancy, thyroid autoantibodies can cross the placenta and either stimulate (thyroid stimulating antibody - TSAb) or block (thyroid blocking antibody - TBAb) the fetal thyroid gland [4]. Maternally transferred antibodies can temporarily affect the thyroid function of the fetus and newborn until they are metabolized. High levels of TSAb transmission are associated with fetal and neonatal thyrotoxicosis, while maternal TBAb can lead to congenital hypothyroidism [12]. The impact on thyroid function in the fetus and newborn depends not only on the type of maternal antibodies but also on their levels. Autoimmune hyperthyroidism can also occur in children born to mothers who were treated for GD in the past and still have detectable circulating TRAbs [13], similar to our patient. TRAbs measurement is not routinely performed in mothers with hyperthyroidism in our hospital, which presents a challenge in identifying those who may develop NH. In our case, the mother’s TRAb levels were not regularly monitored during the 4 years after Iodine-131 radiotherapy, including pregnancy, although she was taking oral euthyrox and had stable thyroid hormone levels within the normal range. Obstetricians and neonatologists often overlook mothers with high-risk factors, leading to misdiagnosis and delayed diagnosis in newborns born to such mothers due to a lack of sufficient understanding in managing these cases.

In China, it is recommended that TRAb should be monitored from 20 to 24 weeks of gestation in pregnant women with a history of Graves’ disease or delivery of a newborn with hyperthyroidism, regardless of whether they have received effective treatment. The American Thyroid Association (ATA) 2016 guidelines [14] recommend that patients with Graves’ disease should be tested for serum TRAb in the first trimester of pregnancy. If TRAb levels are elevated, reexamination should take place at 18–22 and 30–34 weeks of gestation. A TRAb level of ≥ 5 IU/L or > 3 times the upper limit of the reference value indicates a high risk of fetal/neonatal hyperthyroidism. Pregnant women with positive TRAb results should undergo a fetal thyroid ultrasound examination to further evaluate fetal thyroid function. Therefore, for high-risk pregnant women with a history of thyroid disease, it is crucial to detect thyroid function and serum TRAb as early as possible. Close observation of early symptoms of hyperthyroidism is the key to early diagnosis.

The clinical manifestations of NH are non-specific and diverse [8], including tachycardia, irritability, irritability, thrombocytopenia, liver damage, jaundice, shortness of breath, hypoglycemia, hyperhidrosis, premature synostosis, intrauterine growth restriction, growth retardation, goiter, exophthalmia, pulmonary hypertension, hip dysplasia, etc. In severe cases, microcephaly, heart failure, long-term neurodevelopmental delay, and even death may occur. In our study, the neonate presented with growth retardation, tachycardia, cardiac insufficiency, dyspnea, thrombocytopenia, liver injury, and hepatosplenomegaly. Similar cases are often mistaken for intrauterine infection, sepsis, meconium aspiration, and other diseases. The main clinical manifestation in our case is damage to liver and heart function, particularly cholestatic hepatitis [15], which is rare in NH and complicates the clinical diagnosis. Therefore, the crucial aspect in treating liver damage induced by hyperthyroidism is to manage hyperthyroidism itself. Once diagnosed, prompt administration of anti-hyperthyroidism treatment is essential.

Due to the unique nature of the neonatal period, delayed diagnosis and treatment can hurt the physical growth and neurodevelopment of newborns. It is recommended to use ATDs early in neonates showing clinical symptoms of hyperthyroidism, with MMI being the preferred choice. PTU (propylthiouracil) should only be used for a short period in patients experiencing hyperthyroidism crises or severe adverse reactions to MMI. In this case, the patient was administered oral MMI at a dose of 0.5 mg/ kg, Bid. Propranolol (1–2 mg /(kg·d), Bid) can help reduce heart rate and inhibit the conversion of peripheral T4 to T3, especially in patients with increased heart rate. The patient’s heart rate significantly decreased after propranolol was added to her treatment plan. For patients with respiratory and heart failure, it is important to provide respiratory and circulatory support. Short-term glucocorticoids [hydrocortisone 2.5–10 mg /(kg·d), Tid; prednisone 1–2 mg /(kg·d), Bid] can be used to reduce T4 synthesis and peripheral T4 to T3 conversion [16]. In severe cases, intravenous immunoglobulin (1 g/kg for 2 days) may be administered [17]. Adequate caloric supply is crucial in the nutritional support of neonatal hyperthyroidism, and the average course of ATDs treatment for NH is 1 to 3 months. During the initial stage of treatment, thyroid function should be regularly monitored every 1 to 2 weeks to adjust the drug dose. The symptoms gradually disappear with the decrease of TRAb concentration, and treatment can be discontinued when TRAb is negative [16]. In our patient’s case, the total duration of methimazole treatment was 29 days, and the first improvements were FT3 and FT4 levels, with TSH returning to normal levels 13 days after discontinuation of the medication.

Pregnancy complicated with hyperthyroidism has hypermetabolism, increased nerve and muscle excitability, which can lead to uterine contraction and vasospasm, affect placental development, and cause intrauterine growth retardation, low birth weight, premature delivery, asphyxia, and even stillbirth or abortion [7, 18]. In this particular case, the mother had thyroid disease and gave birth to a test-tube baby who was premature and experienced asphyxia after birth. This suggests that maternal thyroid disease can result in complications for the neonate or fetus. Normally, NH is transient and self-limited, resolving within 3–12 weeks. However, in cases of hyperthyroidism crisis, the mortality rate can be as high as 15-20% [6]. Persistent cases are rare, mostly caused by gene mutations such as TSHR and GNAS, which can be inherited dominantly or occur as de novo mutations [6]. Fortunately, in this case, the child was considered to have transient hyperthyroidism, and her thyroid function returned to normal at the age of 57 days without any lasting effects. It is important to regularly monitor the physical and neurodevelopment of children with hyperthyroidism after discharge.

## Conclusion

NH encompasses gynecology, obstetrics, and neonatology. It is important to enhance the monitoring of thyroid function and TRAb in pregnant women with a history of thyroid disease. This will help assess the risk of neonatal hyperthyroidism. Additionally, it is crucial to promptly screen high-risk infants for thyroid function after birth. Early diagnosis and treatment can then be initiated before clinical symptoms worsen, thereby preventing the occurrence of a hyperthyroidism crisis and any potentially serious consequences.

## Data Availability

The datasets generated during and/or analyzed during the current study are available from the corresponding author upon reasonable request.

## References

[1] Stagnaro-Green A, Abalovich M, Alexander E, Azizi F, Mestman J, Negro R, Nixon A, Pearce EN, Soldin OP, Sullivan S (2011). Guidelines of the American thyroid Association for the diagnosis and management of thyroid disease during pregnancy and postpartum. Thyroid: Official Journal of the American Thyroid Association.

[2] McKenzie JM, Zakarija M (1992). Fetal and neonatal hyperthyroidism and hypothyroidism due to maternal TSH receptor antibodies. Thyroid: Official Journal of the American Thyroid Association.

[3] Léger J (2017). Management of fetal and neonatal Graves’ Disease. Hormone Res Paediatrics.

[4] Maximiano C, Silva MR, Carvalho F, Almeida J, Gomes MM, Martins S, Marques O, Estrada A, Pereira A, Antunes A (2021). Follow-up of infants born to mothers with Graves’ disease. Endocrinologia Diabetes Y Nutricion.

[5] Banigé M, Kariyawasam D, Gauthereau V, Luton D, Polak M (2022). Neonatal screening for hyperthyroidism proof of Concept. J Clin Endocrinol Metab.

[6] Samuels SL, Namoc SM, Bauer AJ (2018). Neonatal thyrotoxicosis. Clin Perinatol.

[7] Alves Junior JM, Bernardo WM, Ward LS, Villagelin D (2022). Effect of hyperthyroidism control during pregnancy on maternal and fetal outcome: a systematic review and Meta-analysis. Front Endocrinol.

[8] Bohîlțea RE, Mihai BM, Szini E, Șucaliuc IA, Badiu C. Diagnosis and management of fetal and neonatal thyrotoxicosis. Med (Kaunas Lithuania). 2022;59(1). 10.3390/medicina59010036. 10.3390/medicina59010036 PMC9865749 36676660

[9] Laurberg P, Andersen SL (2016). ENDOCRINOLOGY IN PREGNANCY: pregnancy and the incidence, diagnosing and therapy of Graves’ disease. Eur J Endocrinol.

[10] Dulek H, Vural F, Aka N, Zengin S (2019). The prevalence of thyroid dysfunction and its relationship with perinatal outcomes in pregnant women in the third trimester. North Clin Istanbul.

[11] Lim BH, Raman S, Sivanesaratnam V, Ngan A (1989). Thyrotoxicosis in pregnancy–a six year review. Singapore Med J.

[12] Brown RS, Bellisario RL, Botero D, Fournier L, Abrams CA, Cowger ML, David R, Fort P, Richman RA (1996). Incidence of transient congenital hypothyroidism due to maternal thyrotropin receptor-blocking antibodies in over one million babies. J Clin Endocrinol Metab.

[13] Luz IR, Martins JR, Jerónimo M, Caetano JS, Cardoso R, Dinis I, Mirante A (2020). Neonates born to mothers with Graves’ Disease: 15 year experience of a Pediatric Endocrinology Department. Acta Med Port.

[14] Ross DS, Burch HB, Cooper DS, Greenlee MC, Laurberg P, Maia AL, Rivkees SA, Samuels M, Sosa JA, Stan MN (2016). 2016 American Thyroid Association Guidelines for Diagnosis and management of hyperthyroidism and other causes of thyrotoxicosis. Thyroid: Official Journal of the American Thyroid Association.

[15] Piantanida E, Ippolito S, Gallo D, Masiello E, Premoli P, Cusini C, Rosetti S, Sabatino J, Segato S, Trimarchi F (2020). The interplay between thyroid and liver: implications for clinical practice. J Endocrinol Investig.

[16] van der Kaay DC, Wasserman JD, Palmert MR. Management of Neonates Born to Mothers With Graves’ Disease. Pediatrics. 2016;137(4). 10.1542/peds.2015-1878. 10.1542/peds.2015-1878 26980880

[17] Kurtoğlu S, Özdemir A (2017). Fetal neonatal hyperthyroidism: diagnostic and therapeutic approachment. Turk Pediatri Arsivi.

[18] Kobaly K, Mandel SJ (2019). Hyperthyroidism and pregnancy. Endocrinol Metab Clin North Am.

